# Supplementary material for: Rapid turnover of life-cycle-related genes in the brown algae
Source: Genome Biol. 2019 Feb 14;20:35. doi: 10.1186/s13059-019-1630-6 (PMC6374913; doi:10.1186/s13059-019-1630-6)
Supplement: Supplementary file 3 — Protein alignments, corresponding Gblocks html file and CDS sequences for the lineage conserved gametophyte-biased genes. (ZIP 2109 kb) [file 13059_2019_1630_MOESM3_ESM.zip › Con_GA_alignments/Ec-15_004260.aa_ali.fasta-gb.htm]

Ec-15\_004260.aa.fasta.trans.aa\_ali.fasta


## Gblocks 0.91b Results

Processed file: **Ec-15\_004260.aa.fasta.trans.aa\_ali.fasta**  
Number of sequences: **2**  
Alignment assumed to be: **Protein**  
New number of positions: **308** (selected positions are underlined in blue)

```
                         10        20        30        40        50        60
                 =========+=========+=========+=========+=========+=========+
Ec-15_004260     MSQPSILSFFGKGSSAAPSSAAGKEKIKAKPRKASDRARSAAAGGTAKPTARRRNAVANK
MSTRG.9710.1     ------------------------------------------------------------
                                                                             


                         70        80        90       100       110       120
                 =========+=========+=========+=========+=========+=========+
Ec-15_004260     EKEASAAVDLTAAAPSAPIEAATRALEPEKRQRPSRAVKGDGGGGGDSSNPAAASAATNT
MSTRG.9710.1     ------------------------------------------------------------
                                                                             


                        130       140       150       160       170       180
                 =========+=========+=========+=========+=========+=========+
Ec-15_004260     PNSKRKSRRRKAALAPGSGEDSDVPSPGTDSETSPPALPGGEGRPTKRSRVGGSFGKRSS
MSTRG.9710.1     ------------------------------------------------------------
                                                                             


                        190       200       210       220       230       240
                 =========+=========+=========+=========+=========+=========+
Ec-15_004260     EGNGRDDHTVEEMEEKGVAEDPEPTPPGNPEHTSRLRKKDGKREGVEPNKNGQEGGGNEG
MSTRG.9710.1     ------------------------------------------------------------
                                                                             


                        250       260       270       280       290       300
                 =========+=========+=========+=========+=========+=========+
Ec-15_004260     EGRDANEDTDGEGARVDEGLPFSDAGAPSLRGGEDEGGSEGEGEKDELSEEERGEPKKIN
MSTRG.9710.1     ------------------------------------------------------------
                                                                             


                        310       320       330       340       350       360
                 =========+=========+=========+=========+=========+=========+
Ec-15_004260     RKSPGNTKEEAGAARAGGISQYELERLERIKRNQAFMATLGLATAKPLAAPAATSS----
MSTRG.9710.1     ------------NAEAGGISQYELQRLERIKRNQAFMATLGLATAKPPAASSTGSSSSRS
                              ###########################################    


                        370       380       390       400       410       420
                 =========+=========+=========+=========+=========+=========+
Ec-15_004260     EGTKGRNKNKKRSRPVSRSREKAATVVPVRRSARGRGAEAVDYNEDKQAINAVARAARAA
MSTRG.9710.1     AGGSAKRPNKKRSRPVSRASERAP-AVPVRRSARARGAEAVDYSEDKQAINAIATAARAA
                  ######################   ##################################


                        430       440       450       460       470       480
                 =========+=========+=========+=========+=========+=========+
Ec-15_004260     AAAPAEPEPILEEIDFDDSTVLKYLCGTEANVKDGGGNDSSAEAPNTAAAAAANSQTAAT
MSTRG.9710.1     AAEPPKPDPVLEEVDFDDSTVFKYLC-------EGRGQGDAGDAERSSSAGVT-------
                 ##########################        ################          


                        490       500       510       520       530       540
                 =========+=========+=========+=========+=========+=========+
Ec-15_004260     LTGTGAASAGATVVGLRVIDPD-DPLSASGLSTIYTMHFCGARAAGGGGGGRGAAALPPL
MSTRG.9710.1     ----GPLPGGSRVVGVRVVEPGRAPLSASGLSTIYTMHFCGAGATASGAGG-GRAAAPPL
                     #################   ########################### ########


                        550       560       570       580       590       600
                 =========+=========+=========+=========+=========+=========+
Ec-15_004260     LAAGGKGGVVALFSTRRQQQTGRYENNSNGQEEEEEDERTLMNFKAHKGWVSAVRFLEGG
MSTRG.9710.1     LAAGGKGGIVALFSTQRQE-KGRETGGHNSDEEDEDDERTLMNFKAHKGWVSAVRFLEGG
                 ##################   #######################################


                        610       620       630       640       650       660
                 =========+=========+=========+=========+=========+=========+
Ec-15_004260     GAGAGVGGCRLLTSANDSVVKLWDTSKQHRGTPRLLSTMDDLHPTRKGIFAMDSHGTRVA
MSTRG.9710.1     GSAP---GSRLLSSANDAVVKLWDTSKQHRGLPRLLCTLDDLHPTRKGIFSMDSSGTRVA
                 #      #####################################################


                        670       680       690       700       710       720
                 =========+=========+=========+=========+=========+=========+
Ec-15_004260     TGSKDTTVAIASLRPTGIERDRVLGDPGGSEAFHEKVVKGVSLRDENTVASCGDDSNVRV
MSTRG.9710.1     TGSKX-------------------------------------------------------
                 ####                                                        


                        730       740       750       760       770       780
                 =========+=========+=========+=========+=========+=========+
Ec-15_004260     SDLRSSSSVGVSQRLDGVHGGRACHTVRWHPLDDNRLLSAGLDSTVNLYDLRRLAAPLHV
MSTRG.9710.1     ------------------------------------------------------------
                                                                             


                        790       800       810       820       830       840
                 =========+=========+=========+=========+=========+=========+
Ec-15_004260     FRGHCPYALARPKAIHRPEFLPSGGGNSVNIITCGERSENISMYDAGSGGTVSRGLLGDE
MSTRG.9710.1     ------------------------------------------------------------
                                                                             


                        850       860       870
                 =========+=========+=========+=========
Ec-15_004260     ASALAVGIADWSKGSPEAAGIAAALPGGNVVLLEPVWGS
MSTRG.9710.1     ---------------------------------------
```

```
Parameters used
Minimum Number Of Sequences For A Conserved Position: 2
Minimum Number Of Sequences For A Flanking Position: 2
Maximum Number Of Contiguous Nonconserved Positions: 8
Minimum Length Of A Block: 10
Allowed Gap Positions: None
Use Similarity Matrices: Yes
```

```
Flank positions of the 9 selected block(s)
Flanks: [314  356]  [362  383]  [387  446]  [455  470]  [485  501]  [505  531]  [533  558]  [562  601]  [608  664]  

New number of positions in Ec-15_004260.aa.fasta.trans.aa_ali.fasta-gb:  308  (35% of the original 879 positions)
```
